# The Treatment of Hemotysis

**Published:** 1893-10

**Authors:** 


### THE TREATMENT OF HEMOPTYSIS.

Dr. Fr. Eklund, of Stockholm, (*Therapeutic Gazette*, February, 1893,) severely condemns the common practice of giving cold drinks and small bits of ice to the patient with hemoptysis to swallow. He believes that the irritation of the ends of the pneumogastric nerve in the mucous membrane, by the cold, must result in paroxysms of coughing, as is the experience with many phthisical patients, and that the cold tends to contract the vessels of the stomach and vicinity, leading to higher pressure in the pulmonary area. Instead, he advises lukewarm, mucilaginous drinks. He also advises against the popular practice of giving large amounts of salt dissolved in water, since the fragility of the vessels is increased upon absorption of the sodium chloride and fluid. He suggests that a small ice bag be placed over the bleeding spot, and that quinine be administered as follows :

℞ Sulphate of quinine ........................ ʒj

Extract of ergot. ....................... gr. xxx

M. Make into forty pills, and take one or two pills twice or three times a day.

Or the following may be given :

| | | |
|---|---|---|
| ℞ | Fluid extract of hamamelis | ʒij |
| | Fluid extract of cinchona | ʒij |
| | Extract of liquorice | ʒijss |
| | Distilled water | Oj |

Shake thoroughly, and take a dessertspoonful to a tablespoonful every two or three hours.

He advises that in young persons lead acetate be withheld, for fear of colic; in older persons this remedy may be of service, but should be given in combination with morphine.—*Texas Medical Journal.*

